# Psychological couple-oriented interventions for patients with heart disease and their partners: a scoping review and guidelines for future interventions

**DOI:** 10.3389/fpsyg.2023.1194767

**Published:** 2023-09-29

**Authors:** Giada Rapelli, Emanuele Maria Giusti, Claudia Tarquinio, Giorgia Varallo, Christian Franceschini, Alessandro Musetti, Alessandra Gorini, Gianluca Castelnuovo, Giada Pietrabissa

**Affiliations:** ^1^Department of Medicine and Surgery, University of Parma, Parma, Italy; ^2^EPIMED Research Center, Department of Medicine and Surgery, University of Insubria, Varese, Italy; ^3^Department of Psychology, Catholic University of the Sacred Heart, Milan, Italy; ^4^Department of Biomedical, Metabolic and Neural Sciences, University of Modena and Reggio Emilia, Modena, Italy; ^5^Department of Humanities, Social Sciences and Cultural, University of Parma, Parma, Italy; ^6^Department of Clinical Sciences and Community Health, University of Milan, Milan, Italy; ^7^Istituti Clinici Scientifici Maugeri IRCCS, Milan, Italy; ^8^Istituto Auxologico Italiano IRCCS, Psychology Research Laboratory, Milan, Italy

**Keywords:** heart diseases, partner support, couple-based interventions, psychological interventions, scoping review

## Abstract

**Objective:**

This scoping review aims to provide an accessible summary of available evidence on the efficacy of psychological couple-based interventions among patients with heart disease and their partners focusing on specific aspects and strategies by assessing different emotional and physical cardiac-related outcome measures.

**Methods:**

A literature search was performed in PubMed, Scopus, Medline, PsycINFO, and Web of Science databases using the keywords “heart diseases” and “couple-based intervention.” A literature search using systematic methods was applied. Data were extracted to address the review aims and were presented as a narrative synthesis.

**Results:**

The database search produced 11 studies. Psychological couple-based interventions varied in terms of the type of intervention, personnel, format (group or individual, phone or in person), number of sessions, and duration. Most of the contributions also lacked adequate details on the training of professionals, the contents of the interventions, and the theoretical models on which they were based. Finally, although partners were involved in all the treatment, in most studies, the psychological strategies and outcomes were focused on the patient.

**Conclusion:**

The variability of the psychological couple-based interventions of included studies represents a challenge in summarizing the existing literature. Regarding their impact, psychological interventions for patients with cardiovascular disease and their partners were found to moderately improve patients’ and partners’ outcomes.

## 1. Introduction

Having a heart disease redefines oneself as ill, modifies one’s significant bonds, and requires constant lifestyle changes according to the disease progression (Roger et al., 2020). Furthermore, the management of heart disease is complex and requires constant monitoring of symptoms over time. For this reason, if present, the partner plays an important role by providing both practical and emotional support (Bertoni et al., 2015; Donato et al., 2020). Since romantic relationships play a significant role in people’s lives (Bertoni et al., 2015), it is important to investigate the role of the partner in either helping or hindering the patient’s psychological adjustment to heart disease over the course of the medical treatment.

The role of the partner in cardiovascular disease is central from the acute to the chronic phase of the illness, commonly faced at home (Rapelli et al., 2022). During hospitalization, the presence of a supportive partner can make a difference in terms of the patient’s recovery and psychological wellbeing. By supporting patients’ self-efficacy (Maeda et al., 2013; Rapelli et al., 2022), partners might increase their ability to self-care (George-Levi et al., 2016; Rapelli et al., 2021) even in complex medical situations when an implantable device like the left ventricular assist device is needed (LVAD; Golan et al., 2023; Rapelli et al., 2023). In addition, partners might help to reduce patients’ symptoms of depression and/or anxiety (Sokoreli et al., 2016; Bouchard et al., 2019; Rapelli et al., 2021) or monitor their compliance to complex pharmaceutical therapies, make appointments for follow-ups and accompany the patient to the visits, detect signs of cardiac symptomatology, and be the primary person responsible for the patient’s hyposodic diet (Randall et al., 2009; Rapelli et al., 2020a).

A supportive partner also motivates and helps the patient adopt healthier lifestyle habits–thus reducing cardiovascular risk factors (Maeda et al., 2013; Rapelli et al., 2022) and rates of participation in cardiac rehabilitation programs (Rankin-Esquer et al., 2000).

Conversely, not all forms of support are helpful (Breuer et al., 2017). Indeed, studies have shown that social support may be split into positive and negative forms. Cardiac patients have been the subject of substantial research on positive support, defined as interactions that foster affection (Sebri et al., 2021). On the other hand, scholars paid less attention to negative support, or when the beneficiary of support regards it as unhelpful or feels social limitations by others (Breuer et al., 2017). In fact, patients who feel poorly emotionally supported experience a 41% higher risk of non-compliance with the treatment than those who feel supported by their partner (Leifheit-Limson et al., 2012). Furthermore, perceiving the partner as hostile or overprotective could hinder the patients’ motivation or become a barrier to behavioral changes, thus affecting their health (Fiske et al., 1991; Rapelli et al., 2020b; Bertoni et al., 2022).

Still, partners are not immune to the sense of emptiness and lack of control commonly caused by the disease (Rapelli et al., 2020a), and providing support may be a very stressful and demanding experience for informal caregivers, i.e., those who provide unpaid care to their loved ones (Randall et al., 2009; Bertoni et al., 2015; Rapelli et al., 2020a).

Healthy spouses might present high levels of distress (Randall et al., 2009; Bertoni et al., 2015; Rapelli et al., 2020a) and post-traumatic stress symptoms (Vilchinsky et al., 2017; Fait et al., 2018). Furthermore, partners may refer to absent relational and sexual satisfaction (Bouchard et al., 2019) and perceived low positive dyadic coping, which represents a risk factor for the provision of inadequate support (Rapelli et al., 2021). In addition, it has been speculated that the ability of the spouses of patients with heart disease to be supportive decreases over time, while critical and controlling behaviors increase (Stephens et al., 2006; Rapelli et al., 2022) in the presence of caregiver burden (Luttik et al., 2007).

Since coping with cardiac problems represents, therefore, a dyadic experience rather than proper of the patients (e.g., Rapelli et al., 2021, 2022, 2023; Golan and Vilchinsky, 2023), there is a growing demand for couple-based interventions for heart disease that focus not only on the patients but also on their partners.

For these reasons, this scoping review aims to provide an overview of available evidence on couple-based psychological interventions for coping with heart disease involving both patient and partner by answering the following research questions: (1) What are the main characteristics in terms of the theoretical model, provider, and format of intervention? (2) Which psychological strategies are specifically used in the intervention? (3) Which scoping review outcomes are measured in the short- and long-term?

## 2. Methods

In the present study, the results of a scoping review focused on couple-based interventions for patients with heart diseases and their partners are shown. Data extraction, critical appraisal, and qualitative synthesis were in line with established systematic review and qualitative synthesis methods (Khan et al., 2003).

### 2.1. Search strategy

Searches were conducted in PubMed, Scopus, Medline, PsycINFO, and Web of Science from November to December 2022.

The search strategies combined key terms and Medical Search Headings (MESH) terms based on the PICO (Patient/Population, Intervention, Comparison, and Outcomes) framework as follows: (“CVD” OR “Cardiovascular disease” OR “Cardiac”) AND (“Couple” OR “Dyad” OR “Partner” OR “Caregiver”) AND (“Couple-based intervention” OR “Couple therapy” OR “Couple program”) (Huang et al., 2006). Boolean and truncation operators were used to systematically combine more searched terms and list documents containing variations on search terms, respectively. The search syntax was modified as appropriate for each database.

### 2.2. Inclusion and exclusion criteria

Only original articles that (1) employed couple-based interventions involving both patients and partners; (2) were published in English, (3) examined the impact of couple-based interventions on patients with heart disease and their partners were included. Records were excluded if they (1) considered only biomedical outcome variables, (2) were review articles, single-case studies, mixed-method studies, protocol studies, workplace interventions, theses, or internal reports of gray literature, (3) involved only the patient or their family members as caregivers (e.g., parents, siblings, cousins, etc.). Unpublished works were not considered. No restrictions were set for the date of publication and type of study design.

### 2.3. Study selection

Following the search and exclusion of duplicates, two reviewers (authors GR and CT) independently assessed the eligibility of the articles first on the title and the abstract, and the full text according to the inclusion criteria. Author 3 (CT) resolved disagreements. Following Smith et al. (2011) recommendation, the review team included two people with methodological expertise in conducting systematic reviews (EG and GP) and at least two experts on the topic under review (GR and CT). The reference lists of all selected articles and relevant systematic reviews were manually screened to identify any further references for possible inclusion–but none was found.

**FIGURE 1:**
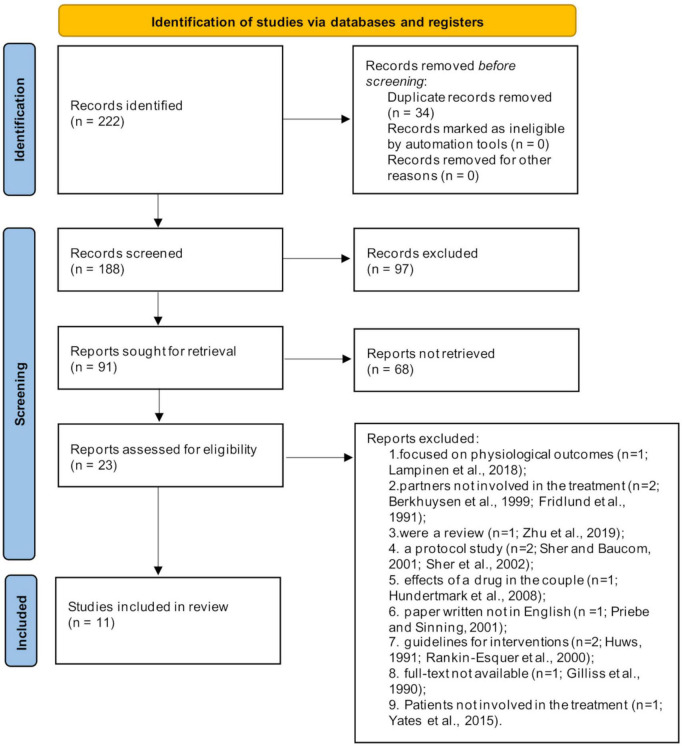
Flow chart diagram.

A search of electronic databases identified 222 reports, of which 165 were excluded based on information from the title and abstract after removing duplicates. The remaining 23 articles were evaluated for inclusion by reviewing their full text, which resulted in the exclusion of 11 records. The flowchart presented in Figure 1 provides step-by-step details of the study selection.

### 2.4. Data extraction and synthesis

Two authors (GR and CT) independently extracted the following data from the included studies: (1) first author and year of publication, (2) country, (3) study aim, (4) study design, (5) sample, (6) measures, (7) characteristics of the intervention, (8) primary outcomes, (9) secondary outcomes, (10) setting, (11) provider, (12) duration of the intervention, (13) follow-up point(s), (14) theoretical approach, (15) intervention approach and format, (16) control group, (17) main findings.

They discussed any discrepancies, and, if necessary, consulted a third author (EG) to reach a final decision (Table 1). Extracted data were collated to produce a narrative summary of couple-based interventions for cardiac patients and their partners. Furthermore, to report all characteristics of studies and interventions the CONsolidated Standards of Reporting Trials 2010 checklist (Consort10; Schulz et al., 2010) and the Template for Intervention Description and Replication checklist (TIDieR; Hoffmann et al., 2014) were used.

**TABLE 1:** Study characteristics.

References	Country	Design	Aim	Sample (*n*) patients: partners	Age (years: Mean; SD; range) patients: partners	Types of CVD	Primary outcomes (measure)	Secondary outcomes (measure)	Follow-up	Control group	Main findings
Daugherty et al., 2002	USA	Qualitative interview study with focus group	To evaluate a couple-based intervention facilitating partners mutual coping during the cardiac acute phase	15:15	NR	Chronic or acute cardiac illness	Behavior changes (e.g., cardiac risk-factor reduction).	NR	NR	No CG	The single-session intervention promotes the mutual knowledge of partners. The intervention is perceived as useful for couples. Common problems of couples during the intervention include overprotection, criticism, feelings of responsibility and guilt, fear, and greater awareness about the support provided to the patient and the importance to change it when ineffective. It should be noted that the training provided to nursing staff is perceived as inadequate. The complexity, logistic barriers, and time constraints limited the technique and the skills of nursing staff in carrying out psychosocial interventions.
Dinesen et al., 2019	Denmark	Qualitative interview study	To explore the experiences of cardiac patients and their partners of participating in the Teledialog Telerehabilitation Program (TTP consisted of a digital rehabilitation plan, transmission of health data from patient’s home to hospital and health care center, and an interactive Web portal with information and training videos.)	14:12	NR	acute coronary syndrome, heart failure, or coronary artery bypass surgery/valve surgery	Experiences of the cardiac patients and their partners about intervention program	NR	Baseline, 12-weeks follow-up	No CG	Both patients and partners express that the Active Heart site is a useful tool for educating patients in the rehabilitation process and that it provides relevant information about heart disease, symptoms, and lifestyle changes. Then, telerehabilitation gives them an overview of goals, plans, and appointments, creating a greater sense of coherence in the rehabilitation process and fostering patient autonomy because they feel more involved in making personal decisions about the rehabilitation process. The partners evaluate the telematic rehabilitation plan as very useful to facilitate understanding and consistency in the rehabilitation process for both themselves and the patient, thus creating a greater feeling of safety. Some partners, however, take on too many responsibilities during their participation in the rehabilitation program and find it difficult to orient themselves between the desire to be involved, to be overprotective toward the patient and at the same time remain an equal partner in the rehabilitation process. It turns out that partners have become an important support resource in everyday life for patients, motivating them to continue changes in their training lifestyle alone. Many patients report a lack of sense of belonging to a therapeutic community.
Dracup et al., 1984	USA	RCT	To evaluate the effect of increased involvement of spouses within the context of a group counseling intervention on the compliance of cardiac patients	58:58 IG1: *N* = 17 cardiac patients and their partners IG2: *N* = 22 cardiac patients and their partners CG: *N* = 19 cardiac patients and their partners	57.00; NR; NR: NR; NR; NR	CABG; Myocardial infarction	Compliance to a cardiac risk-factor regimen: smoking, blood pressure, body weight, weekly exercise	NR	Baseline, 10 weeks and 6 months follow-ups	TAU	Counseling intervention, based on the symbolic interactionism approach, reports greater positive effects on the compliance of the heart risk factors: smoking, blood pressure, body weight, and weekly exercise. In particular, smoking has not changed over time, in fact, the behavior related to smoking has not been significantly different over time between groups for both patients and partners. The experimental groups have significantly lower blood pressure recorded at 6 months in the follow-up. The largest decrease is in IG2. The results show a significant difference between the groups in body weight: in fact, both IG1 and IG2 obtained and maintain long-term weight loss compared to the TAU, in which body weight increases in the follow-up by 6 months. Patients in IG1 have greater compliance on cardiac risk factors, but participation in a cardiac rehabilitation program is not, in itself, sufficient to influence long-term compliance. Then, by results, the role of family members may have an enabling or inhibiting effect on patient compliance; they may or may not apply a reason for complying with a specific system of conduct.
Gortner et al., 1988	USA	RCT	1. To test the effectiveness of nursing interventions designed to improve post-hospital recovery and rehabilitation at home; 2. to assess the impact of valve replacement and bypass surgery on the health of the family unit	67:67 IG: *N* = 32 cardiac patients and their partners CG: *N* = 35 cardiac patients and their partners	61.5; NR; NR: NR; NR; NR	CABG; valve replacement surgery	Self-efficacy (*ad hoc* Gortner et al., 1988) Family functioning (The family APGAR); Smilkstein, 1978 Family Resources (FIRM); McCubbin et al., 1987 Marital satisfaction (MAS); Locke and Wallace, 1959 Mood state (POMS); McNair et al., 1971 Activities (*ad hoc* self-report)	NR	3 months and 6 months post-discharge	Patients of CG assist for the entire intervention program, except the counseling session and telephone session following third monitoring program	IG > CG for self-efficacy in the lifting of objects at 3 months (*t* = 2.19, *p* = 0,034), and CG > IG for tolerance of stress and anger (emotional distress) at 3 months (*t* = 2.90, *p* = 0,007). No significant differences between groups for partners. At 6-months follow-up the differences are not, statistically significant. There were not significant differences on POMS and significant differences between groups for partners. For the family functioning APGAR, FIRM, and MAS no significant differences were observed between patients and partners of IG and those of CG at 3- and 6-months follow-ups. People over the age of 70 have a higher perception of self-efficacy effort and report significantly lower levels of hostility and depression compared to those in their 50 s. With regard to family functioning and family resources, there are not significant differences in measures between groups; however, the partners report lower levels compared to patients.
Hartford et al., 2002	Canada	RCT	To determine the effectiveness of an information and support telephone intervention for reducing anxiety in patients who have undergone CAGB surgery and their partners.	131:131 IG: *N* = 63 cardiac patients and their partners CG: *N* = 68 cardiac patients and their partners	Patient age: (IG: *M* = 62.7; SD = 9.1); (CG: *M* = 63.0; SD = 8.2) Partner age: (IG: *M* = 59.9; SD = 9.9); (IG: *M* = 60.8; SD = 9.4)	CAD	Anxiety levels after surgery (BAI; Gill and Murkin, 1996)	NR	3 days, 4 and 8 weeks post-discharge	TAU	Anxiety evolves from moderate/severe levels at the baseline until the last assessment; so, anxiety can be resolved over time. No significant differences between groups for both patients and partners in anxiety levels. It is significantly lower in the IG than in the CG at day 2 at home. Partners always have lower anxiety than patients. A more sustained decrease in anxiety in the partner IG is found at both day 2 and week 4.
Johnston et al., 1999	Scotland	RCT	To evaluate the effectiveness of a cardiac counseling and rehabilitation program run by a nurse for patients and their partners on a comprehensive range of psychological and functional outcomes compared with TAU	100:72 IG1: 29 cardiac patients and 19 partners IG2: 38 cardiac patients and 29 partners CG: 33 cardiac patients and 24 partners	Patient age: (IG1: *M* = 57.34; SD = 8.92); (IG2: *M* = 54.05; SD = 7.62); (CG:*M* = 57.00; SD = 9.04) Partner age: (IG1: *M* = 52.20; SD = 9.52); (IG2: *M* = 50.33; SD = 11.49); (CG: *M* = 52.59; SD = 10.53)	MI	Knowledge of the disease and rehabilitation process about pharmacological adherence, diet, and physical activity (*ad hoc*); Johnston et al., 1999 Anxiety and depression (HADS; Zigmond and Snaith, 1983) Satisfaction of care (*ad hoc*; Johnston et al., 1999) Limitations and return to normal activities (FLP; Patrick and Peach, 1989)	NR	Baseline; at discharge; 2-weeks follow-up after discharge; 2-, 6- and 12-months follow-up	TAU	Patients and partners in IG1 and IG2 reported higher levels of knowledge than CG: specifically, patients report higher levels of correct information and ideas and lower levels of uncertain information [Patients: *correct*, *F*(4,166) = 7.94, *p* = 0.0005; *misconceptions*, *F*(4,166) = 4.73, *p* = 0.001; *uncertainty*, *F*(4,166) = 4.04, *p* = 0.004]; Partners. [*correct*, *F*(4,98) = 10.35, *p* = 0.0005; *misconceptions*, *F*(4,98) = 4.01, *p* = 0.005; *uncertainty*, *F*(3.2,78.8) = 5.28, *p* = 0.002]. On depression, patients and partners of IG1 had lower levels than the CG at 6 months; whereas the IG2 has lower levels than the CG at 2- and 6-months follow-up [Patients: *F*(5.9,256.6) = 3.43, *p* = 0.003]; Partners: [*F*(5.7,134.3) = 3.08 g, *p* = 0.008]. On anxiety, patients and partners of IG1 had lower scores than the CG at discharge, and at 2 months and the IG2 had lower levels than the CG at 2-, 6-, and 12-months follow-ups (Patients: [*F*(7.0,302.7) = 2.32 g, *p* = 0.03]; Partners: [*F*(5.7,133.1) 5 2.28, *p* = 0.043]. The IG2 has lower anxiety than IG1 at 2- and 6-months follow-up. On satisfaction with care IG1 and IG2 have higher levels than CG. For satisfaction with care patients and partners of IGs showed higher levels of it compared with CG at 2-months follow-up (Patients: [*F*(2,85) = 9.94, *p* < 0.0005]; Partners: [*F*(2,55) = 32.12, *p* < 0.0005]. For FLP patients of IGs showed lower level of it compared with CG [*total*, *F*(2,85) = 8.77, *p* < 0.0005; *physical*, *F*(2,89) = 8.92, *p* < 0.0005; *psychosocial*, *F*(2,89) = 9.03, *p* < 0.0005].
Lenz and Perkins, 2000	USA	RCT	To assess the impact of a staged, postoperative psychoeducational intervention for CABG patients and their families on patient and family member outcomes	45:45 IG: 22 cardiac patients and their partners CG: 23 cardiac patients and their partners	60.2; 10.6; NR: NR; NR; NR	CABG	Depression (CES-D; Radloff, 1977) Physical State (COOP charts; Nelson et al., 1987) Patient Satisfaction with nursing staff (*ad hoc*; Jacox et al., 1997)	The physical and emotional state of the family caregiver	3–4 days post-surgery; 2, 4, 6, and 12 weeks post-discharge	Pre-discharge videotap, at least 1 home visit	No significant differences between groups for patients regarding CES-D (*F*1,34 = 0.01, *p* < 0.91). COOP (overall: *F*1,36 = 0.09, *p* < 0.67; physical: *F*1,35 = 0.24, *p* < 0.63; emotional: *F*1,36 = 0.24, *p* < 0.63). On satisfaction with nursing care patients of IG reported higher levels than the CG at 3-months follow-up, but not significantly (*F*1,35 = 1.87, *p* < 0.18). For partners, there was no significant difference between IG and CG, for COOP (*F*1,31 = 0.62, *p* = 0.44) and depression, but their depressive symptoms decreased over time (*F*1,32 = 0.48, *p* = 0.49).
Sher et al., 2014	USA	RCT	To evaluate the effectiveness of a patient and partner approach to behavior change compared with a patient-only approach on such factors as exercise, nutrition, and medication adherence	80:80 IG couple condition: 41 cardiac patients and their partners CG individual condition: 39 cardiac patients	60.16; 10.2; NR: 56.87; 11.7; NR	CAD; MI; CABG; angioplasty	Weight and physical measurements (BMI) Physical activity (YPAS; DiPietro et al., 1993) Adhesion to drugs (computerized MEMS) Patient Dyadic Adjustment (DAS; Spanier, 1976)	- Partner Dyadic Adjustment (DAS; Spanier, 1976)	Baseline, 6-, 12- and 18-months follow-ups	Individual Group training consisted of up to 10 patients and a therapist. Group discussion focused on the didactic topic of the day as well as personal reflections related to the patient’s health, with the therapist again serving as a resource person.	IG has significantly higher YPAS level than CG at 18-month follow-up (*B* = −0.06 [95% CI: −0.119 to −0.001], *p* = 0.043, d = 0.82; large effect size). There is also an interaction between marital satisfaction and treatment condition: patients in CG who reported higher levels of marital distress did not maintain their physical activity gains by the end of treatment, while both distressed and non-distressed patients in IG exhibited accelerating gains throughout treatment. There are no significant changes in medication adherence across groups (B = −0.021 [95% CI: −0.393 to 0.351], *p* = 0.27), but patients in CG showed a 9% relative decrease across time. There is an interaction between baseline marital satisfaction and treatment condition: patients in CG who reported lower levels of initial marital satisfaction showed deterioration in marital satisfaction, while non-satisfied patients in IG showed improvement over time [*B* = 0.0002 (95% CI: 0.0004 to 0), *p* = 0.050]. There were no significant effects for nutritional outcomes and weight loss variables (such as BMI) across time or between treatment conditions.
Stewart et al., 2001	Canada	Qualitative interview study	To evaluate the support group intervention for cardiac patients and their partners focusing on what factors influenced the impact of this support group intervention	14:14	57; NR; NR: 56; NR; NR	MI	Social support	NR	NR	No CG	The social comparison gives benefits to the group: couples normalize their experience and felt understood and reassured. The couple perceives the group as a safe place to expose their experiences and where they could learn. Information support has reduced uncertainty in couples about the activities to be followed in rehabilitation; emotional support has reassured and given hope to couples. The intervention also greatly improves communication, understanding, and acceptance within the couple; it promotes better lifestyle adaptation and a change in coping strategies to make the stressful situation worse; it increases the couple’s sense of control and marital quality.
Thompson, 1989	Great Britain	RCT	To evaluate the efficacy of a program of nursing support and education focusing on levels of anxiety and depression reported by first MI male patients and their partners, throughout the patient’s stay in hospital.	30:30 IG: 30 patients and their partners CG: 30 patients and their partners	Patient age: (IG: *M* = 58.8; SD = 7.4); (CG: *M* = 55.9; SD = 7.2) Partner age: (IG: *M* = 50.6; SD = 8.2); (CG: *M* = 54.6; SD = 8.2)	MI	-Anxiety and Depression (HADS; Zigmond and Snaith, 1983)	NR	Baseline, 5 days after discharge, 1-, 3-, and 6 months follow-ups	TAU	The mean scores for patient anxiety in the IG were statistically significantly decreased in comparison to the CG (*P* < *0.0005*), as were the mean scores for patient depression *(P* = *0.01).* The mean scores for partner anxiety in the IG were statistically significantly decreased in comparison to the CG *(P* = O.O1), but the difference in mean scores for depression in partners was not statistically significant *(P* > 0.10).
Tulloch et al., 2021	Canada	Pre-post study	To assess the clinical benefit of an attachment-based relationship enhancement program for couples in which 1 partner has CVD, on relationship quality, mental health, and quality of life	39:39	59.18; 9.2; NR: 56.4; 9.5; NR	Hypertension, coronary artery disease, cardiomyopathy, congenital heart disease, valvular heart disease, and arrhythmias	Patient and Partner Relationship Quality (DAS; Spanier, 1976), Patient and Partner Couple Satisfaction (CSI; Funk and Rogge, 2007) Anxiety and Depression (HADS; Zigmond and Snaith, 1983), Quality of life (SF-36). distinguishing QoL MCS (quality of life mental component) and QoL MCS (quality of life mental physical)	Participant satisfaction (5-point Likert scale)	8 weeks	No CG	DAS patient: *M* change = + 7.5 points; *t*(28) = 3.60, *p* = 0.001; DAS partner: *M* change = + 8.4 points; *t*(28) = 4.46, *p* < 0.001; HADS-D patient: *M* change = −1.9 points; *t*(28) = 3.22, *p* < 0.003; HADS-D partner: *M* change = −1.8 points; *t*(28) = 3.35, *p* < 0.002). Clinically significant changes were observed on the CSI for both parties (patient: *M* change = + 3.0 points; *t*(28) = 1.16, *p* = 0.25; partner: *M* change = + 4.1 points; *t*(28) = 1.4, *p* < 0.170). Patients also reported statistically significant changes in QoL-MCS scores (*M* change = + 3.4 points; *t*(28) = 2.20, *p* = 0.034), whereas partners reported clinically and statistically significant changes in anxiety (*M* change = - 2.1 points; *t*(28) = 3.50, *p* < 0.001). No significant changes were noted for QoL-PCS for patients or partners.

APGAR, adaptability, partnership, growth, affection, and resolve; BAI, Beck Anxiety Inventory; BMI, body mass index; CABG, coronary artery bypass graft; CAD, coronary artery disease; CCU, Coronary Care Unit; CES-D, Center for Epidemiologic Depression Scale; CG, control group; CSI, Couple Satisfaction Index; CHARMS, Cardiac Health and Relationship Management and Sexuality; CVD, cardiovascular diseases; DAS, Dyadic Adjustment Scale; DBS, Decisional Balance Scales; FIRM, Family Inventory of Resources for Management; IG, intervention group; MAS, marital adjustment test; FLP, Functional Limitations profile; HADS, Hospital Anxiety and Depression Scale; HCCQ, Health Care Climate Questionnaire; MI, myocardial infarction; POMS, profile of mood states; RMICS, Revised Marital Interaction Coding System; SF-36, Medical Outcomes Survey Short Form-36; TRSQ, Treatment Self-regulation Questionnaire; TTP, Teledialog Telerehabilitation Program; VAS, Visual Analogue Scale; YPAS, Young Person’s Advisory Service.

## 3. Results

### 3.1. Characteristics of the included studies

The studies included in this review are described in Table 1.

The selected articles were published from Dracup et al. (1984) to Tulloch et al. (2021), and were conducted in the USA (Dracup et al., 1984; Gortner et al., 1988; Lenz and Perkins, 2000; Daugherty et al., 2002; Sher et al., 2014), Denmark (Dinesen et al., 2019), Canada (Stewart et al., 2001; Hartford et al., 2002; Tulloch et al., 2021), Scotland (Johnston et al., 1999), and Great Britain (Thompson, 1989). Three studies employed a qualitative method (Stewart et al., 2001; Daugherty et al., 2002; Dinesen et al., 2019), and six studies employed a randomized controlled trial (RCT) design (Gortner et al., 1988; Thompson, 1989; Johnston et al., 1999; Lenz and Perkins, 2000; Hartford et al., 2002; Sher et al., 2014). In the study by Tulloch et al. (2021), a pre-post-study design was employed.

### 3.2. Description of participants

Selected contributions included a total of 665 patients with cardiovascular disease (CVD) and 602 partners of both genders. The sample size varied from a minimum of 14 patients and 12 partners (Dinesen et al., 2019) to a maximum of 72 patients and their partners (Hartford et al., 2002) across studies. The mean age was 58.62 years for the patients and 57.40 years for the partners involved in these 8 studies (Dracup et al., 1984; Gortner et al., 1988; Thompson, 1989; Johnston et al., 1999; Stewart et al., 2001; Hartford et al., 2002; Dinesen et al., 2019; Tulloch et al., 2021). Three studies did not report patients’ and partners’ ages (Lenz and Perkins, 2000; Daugherty et al., 2002; Sher et al., 2014). In 8 studies, patients suffered from acute or chronic cardiac illness (Dracup et al., 1984; Thompson, 1989; Johnston et al., 1999; Stewart et al., 2001; Daugherty et al., 2002; Sher et al., 2014; Dinesen et al., 2019; Tulloch et al., 2021), while three records included cardiac surgery patients (Gortner et al., 1988; Lenz and Perkins, 2000; Hartford et al., 2002).

### 3.3. Description of intervention

The main characteristics of the interventions were extensively reported in Supplementary material 1 using the CONsolidated Standards of Reporting Trials 2010 checklist (Consort10; Schulz et al., 2010) and the Template for Intervention Description and Replication checklist (TIDieR; Hoffmann et al., 2014), and were summarized in Table 2.

**TABLE 2:** Characteristics of the intervention.

References	Setting	Provider	Duration of intervention	Theoretical approach	Intervention approach	Brief description of the intervention
Daugherty et al., 2002	Clinical setting	Nurses	A single session of 3 h	Social support (Cobb, 1976); emotional theory (Cobb, 1976).	Psychoeducational	Couples assist focus group meetings and individual interviews aimed to talk about the benefits of social support and the importance of making lifestyle changes together, to modify ineffective behaviors, like criticism and overprotection (role-playing session and discussion about expectations and the importance of self-monitoring) and to decrease barriers
Dinesen et al., 2019	Couple home setting	Nurse and a professional with psychological background	12 weeks	Community of practice approach (Lave and Wenger, 1991); self-determination theory (Deci and Ryan, 2000);	Psychoeducational	Active Heart is a telerehabilitation program with an interactive Web portal with information on heart functions, heart diseases, and symptoms, videos with instructions on exercises, and brief rehabilitation narratives by patients and relatives. There was also a Web forum enabling patients to communicate with each other. The Web forum was moderated by a nurse. Individual and group-based education within the following themes: self-management, physical activity, nutritional counseling, medications, psychosocial support, and managing a new lifestyle
Dracup et al., 1984	Outpatient cardiac rehabilitation centers	Two facilitators’ nurses, one the coordinator of the cardiac rehabilitation program at each center and the second a nurse with a master’s degree in nursing and special expertise in group dynamics	10 weekly sessions of 90 min. each	Interactionist role theory (Dracup and Meleis, 1982)	Psychoeducational	IG1 and IG2, are in-person group counseling programs on problem-solving. The difference between experimental conditions is that the IG1 is a couple-based intervention delivered in person and in a group format, in the IG2 only patients participate in the group intervention.
Gortner et al., 1988	3 hospitals	Nurses	NR	Self-efficacy theory (Bandura, 1986); Double ABCX Model (McCubbin and Patterson, 2014)	Psychoeducational	Couples assist in-hospital teaching program to encourage exercise, diet adherence, and surgical recovery; counseling program to provide families with anticipatory guidance on recovery at home and common emotional responses in the immediate post-discharge period; monitoring program to assess self-efficacy level. The couple teaching program is followed by couple telephone support for 8 weeks
Hartford et al., 2002	Cardiology hospital unit and patients’ home	Nurses	6 sessions on days 1, 2, and 4, week 1, 2, and 7 (duration: 20–60 min.)	Emotional distress (Endler and Parker, 1990)	Psychoeducational	Couple education and support delivered in person at discharge as inpatients and 6 telephone calls over 7 weeks (In person first, then phone). The intervention consists of information and support to assist patients and partners in meeting their needs (dyad format). Topics are: (1) graded activity and exercise, (2) pain, (3) psychosocial problems, (4) medications, (5) diet, (6) constipation, (7) smoking cessation, (8) cardiac disease, (9) cardiovascular risk factors, (10) diagnostic tests, and (11) sleep
Johnston et al., 1999	NR Coronary Intensive Care Unit	Nurse counselor	IG1: Up to 5; IG2: up to 86.	Emotional distress (Endler and Parker, 1990)	Psychoeducational	Information, counseling and stress management (in person format) IG1: receives cardiac rehabilitation from a nurse counselor as inpatient IG2: inpatient and outpatient because patients receive the same cardiac rehabilitation as the IG1, but with additional sessions continuing up to 6-weeks after discharge from hospital (Extended program).
Lenz and Perkins, 2000	NR	Cardiac nurses (individual session), researchers (phone sessions) and psychiatric nurses (group session)	12 sessions (Daily and then bi-weekly)	Emotional distress (Endler and Parker, 1990)	Psychoeducational	TAU + Dyad counseling, support and problem solving for 12 sessions. Some sessions conducted over the telephone. Some sessions conducted in group format. Patients assist standard discharge care. Pairs assist dyadic psycho-educational counseling, support and problem solving for 12 sessions; some sessions conducted over the telephone and some of them conducted in a group format in person.
Sher et al., 2014	NR	Therapist	18-months (12 weekly sessions followed by 6 alternate week sessions over a total of 24 weeks)	Cognitive behavioral relationship couples therapy theory (CBCT, Baucom et al., 1998); Theory of Self-Determination (Deci and Ryan, 2000); Transtheoretical Model of Behavioral Change (Prochaska and DiClemente, 2005)	Psychoeducational	IG participated in a group format for 18 sessions focused on the educational component plus communication skills training, motivation discussions, and relationship issues. The group is based on couple-level discussions of the day’s topic as well as practice time for the communication skills being taught. Therapists served as a source for the couples’ discussion, observing and making suggestions for the content of the discussion as well as the process. The relationship content in the couples intervention instructed and encouraged patients and their partners to collaborate on making behavioral and relationship changes.
Stewart et al., 2001	NR	Professional and peer supporters	12 weeks of 1 h	Coping (Lazarus, 1974; Folkman, 1984); Social Support (Bloom, 1990)	Psychoeducational	Patients and partners assist support group intervention, for 1 h weekly for 12 weeks. Support groups are designed to convey support specific for stressful situations encountered by MI survivor and his/her partner. Weekly the topics change based on common stressors experienced. Group discussions are augmented by varied techniques and resources depending on the topic (e.g., role-playing, invited consultant or guest speaker, focused group discussion, guided group exercises, etc.)
Thompson, 1989	CCU hospital unit	Nurses	4 sessions of 30 min each one	Emotional distress (Endler and Parker, 1990)	Psychoeducational	Couple in-hospital education and counseling for 4 sessions (in person). Structured support and education package of 4 sessions focused on the patient’s and wife’s reactions to the feelings toward the heart attack.
Tulloch et al., 2021	Cardiac center	Clinical psychologists	8 weekly 2-h sessions	Emotionally focused therapy (EFT; Wiebe and Johnson, 2016; Beasley and Ager, 2019), Attachment theory (Bowlby, 1969, 1973)	Psychoeducational	In-person group with couples. Participants are guided through seven conversations, based on EFT principles, in which they learn to communicate their need for connection and reassurance. The focus is on CVD and healthy coping together. Partners become adept at recognizing problematic relationship patterns and rectifying them through a series of structured conversations that involve identifying the impact of CVD on their relationship, acknowledging their fears and longings in the aftermath of CVD, healing emotional injuries, and discussing their sexuality in light of their health.

#### 3.3.1. Intervention group

##### 3.3.1.1. The format of the intervention

In all the selected studies, the treatment aimed at providing heart disease-related information to both patients and their informal caregivers–with the main aim to address treatment expectations, and ambivalence toward behavioral change, as well as to define goals and increase the patient-partner dyad’s adherence to treatment recommendations.

The intervention was delivered through regular in-person focus group meetings in 6 out of 11 studies (Dracup et al., 1984; Lenz and Perkins, 2000; Stewart et al., 2001; Daugherty et al., 2002; Sher et al., 2014; Tulloch et al., 2021). The number of intervention sessions ranged from one (Daugherty et al., 2002) to 18 (Sher et al., 2014). In particular, in the study by Daugherty et al. (2002) the intervention group (IG) consisted in talking about the benefits of social support and of the importance of making lifestyle changes together as partners to modify ineffective behaviors (i.e., criticism and overprotection) using discussion and role-playing. In the study by Dracup et al. (1984), the impact of 10 individual sessions with patients only was compared with a couple-based intervention of equal length. In both IGs, a counseling program on problem-solving was delivered.

In the study by Lenz and Perkins (2000), only partners participated in the focus groups, alternated by telephone sessions. In the study by Sher et al. (2014), couples-assisted focus training groups on behavioral change were provided. In the study by Stewart et al. (2001) patients and partners support group interventions were delivered. In the study by Tulloch et al. (2021), patients and partners participated in an in-person group focused on disease management.

Four interventions out of 11 (Gortner et al., 1988; Thompson, 1989; Johnston et al., 1999; Hartford et al., 2002) were in-person individual/(couple) education programs followed by weekly telephone support sessions provided by nurses for 7 (Hartford et al., 2002) or 8 weeks (Gortner et al., 1988). The number of in-person sessions ranged from one (Gortner et al., 1988) to 6 (Hartford et al., 2002). Specifically, the intervention delivered in the study by Gortner et al. (1988) focused on promoting self-efficacy, emotional distress, increased physical activity, and adherence to diet among patients. In the study by Hartford et al. (2002) the intervention consisted of information and support to assist patients and partners in meeting their needs. Johnston et al. (1999) compared 2 types of interventions: inpatient (IG1) vs. inpatient plus outpatients (IG2) counseling for stress management. One intervention was conducted online (Dinesen et al., 2019) and focused on self-management, physical activity, nutritional counseling, and adherence to medications, besides providing psychosocial support.

##### 3.3.1.2. The psychological strategies used in the intervention

Only 2 out of the 11 contributions detailed the psychological strategies employed in the intervention (Stewart et al., 2001; Tulloch et al., 2021). In particular, in the study by Stewart et al. (2001) the topics changed weekly based on experienced common stressors. Group discussions were enriched by varied techniques and resources depending on the topic (e.g., case study scenarios focused on a couple coping with a recent myocardial infarction (MI), role-plays, invited consultant or guest speakers, focus group discussions, guided group exercises–for example, weekly diaries about the perceived importance of a given topic, etc.). In the study by Tulloch et al. (2021), couples were guided through seven conversations in which they learned to communicate their need for connection and reassurance, recognize problematic relationship patterns, and rectify them through a series of structured conversations facilitated by different therapeutic strategies including (a) recognize and name emotional states (known as “symbolization”), (b) engage in direct expressions of vulnerability and need (“enactments”), and (c) respond to clear manifestations of vulnerability and a desire for connection (“empathic attunement through enactment”).

##### 3.3.1.3. The provider of the intervention

The majority of the psychological interventions were provided by one or more trained nurses in 7 interventions (Dracup et al., 1984; Gortner et al., 1988; Thompson, 1989; Johnston et al., 1999; Lenz and Perkins, 2000; Daugherty et al., 2002; Hartford et al., 2002); while in two studies to lead the intervention was a therapist or clinical psychologists (Sher et al., 2014; Tulloch et al., 2021), and in the study by Dinesen et al. (2019) a nurse and a professional with psychological background. Only in one study (Stewart et al., 2001), professionals from various disciplines all working regularly with persons with cardiac disease and/or community-based client groups, and peer supporters (couples in which one spouse was at least 1-year post-MI) conducted the intervention.

##### 3.3.1.4. The theoretical background of the intervention

The theoretical background of the intervention varied across the studies. Four studies (Thompson, 1989; Johnston et al., 1999; Lenz and Perkins, 2000; Hartford et al., 2002) referred to the emotional distress theory (Endler and Parker, 1990). Two studies (Stewart et al., 2001; Daugherty et al., 2002) referred to the social support theory (Cobb, 1976; Bloom, 1990) and the self-determination theory of Deci and Ryan (2000), Sher et al. (2014), and Dinesen et al. (2019), respectively. Other theoretical backgrounds informing the delivered interventions were: the social support theory (Cobb, 1976) in the study by Daugherty et al. (2002), the theory of coping (Lazarus, 1974; Folkman, 1984) in Stewart et al.’s (2001) contribution, the community of practice approach (Lave and Wenger, 1991) in the study by Dinesen et al. (2019), the transtheoretical model of behavioral change (Prochaska and DiClemente, 2005) in the contribution of Sher et al. (2014), and the interactionist role theory (Dracup and Meleis, 1982) in Dracup et al.’s (1984) study. Moreover, Gortner et al. (1988) referred to both the self-efficacy theory (Bandura, 1986) and the double ABCX Model (McCubbin and Patterson, 2014), while Tulloch et al. (2021) referred to the emotionally focused therapy (EFT; Wiebe and Johnson, 2016; Beasley and Ager, 2019) and the attachment theory (Bowlby, 1969, 1973).

#### 3.3.2. Control group

Four studies compared the IG with the treatment-as-usual (TAU) condition (Dracup et al., 1984; Thompson, 1989; Johnston et al., 1999; Hartford et al., 2002). Furthermore, educational counseling group or individual sessions focused on increasing awareness of the benefits of a healthy lifestyle were used as controls in three contributions (Gortner et al., 1988; Lenz and Perkins, 2000; Sher et al., 2014), while four records did not include any control groups (CGs) (Stewart et al., 2001; Daugherty et al., 2002; Dinesen et al., 2019; Tulloch et al., 2021).

### 3.4. Outcomes and effects of the intervention across time-points

#### 3.4.1. Study duration

Study duration ranged from 2 months (Hartford et al., 2002; Tulloch et al., 2021) to 18 months (Sher et al., 2014). In three studies the intervention had a total duration of 3 months (Lenz and Perkins, 2000; Stewart et al., 2001; Dinesen et al., 2019), in three studies the intervention had a total duration of 6 months (Dracup et al., 1984; Gortner et al., 1988; Thompson, 1989), and only in one study the intervention had a total duration of 12 months (Johnston et al., 1999).

#### 3.4.2. Outcomes

**FIGURE 2:**
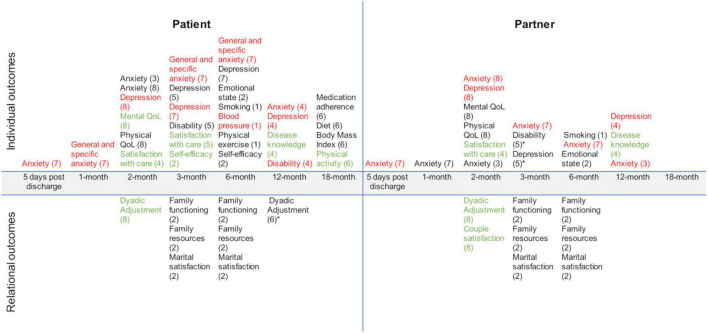
Primary and secondary outcomes across included studies. 1 = Dracup et al., 1984; 2 = Gortner et al., 1988; 3 = Hartford et al., 2002; 4 = Johnston et al., 1999; 5 = Lenz and Perkins, 2000; 6 = Sher et al., 2014; 7 = Thompson, 1989; 8 = Tulloch et al., 2021. In black the non-significant outcomes; In red the decreased significant outcomes, and in green the increased significant outcomes. *Secondary outcomes.

Significant and non-significant effects for both patients and partners are reported in Figure 2. Furthermore, in Supplementary material 2 an extensive summary of the primary and secondary outcomes of the included studies is presented. Briefly, this scoping review showed that couple-based interventions that involved both patients and partners focused on individual outcomes only, relational outcomes only, or both. One out of the 11 selected studies focused on patient individual outcomes only (Sher et al., 2014) not considering partner individual and relational outcomes. Four out of 11 studies focused on patient and partner individual outcomes (Dracup et al., 1984; Thompson, 1989; Johnston et al., 1999; Hartford et al., 2002). Three out of 11 studies focused on patient individual and relational outcomes (Gortner et al., 1988; Sher et al., 2014; Tulloch et al., 2021). Two studies out of 11 focused on patient and partner individual and relational outcomes (Gortner et al., 1988; Tulloch et al., 2021).

As shown in Figure 2, the primary outcome is variable across the included studies. Regarding the patient’s primary outcomes: anxiety was measured in 4 studies (Thompson, 1989; Johnston et al., 1999; Hartford et al., 2002; Tulloch et al., 2021), depression was measured in 4 studies (Thompson, 1989; Johnston et al., 1999; Lenz and Perkins, 2000; Tulloch et al., 2021), disease knowledge was measured in 3 studies (Dracup et al., 1984; Johnston et al., 1999; Sher et al., 2014), physical status was measured in 3 studies (Johnston et al., 1999; Lenz and Perkins, 2000; Sher et al., 2014), satisfaction with care was measured in 4 studies (Johnston et al., 1999; Lenz and Perkins, 2000; Daugherty et al., 2002; Dinesen et al., 2019), mood states were measured in one study (Gortner et al., 1988), quality of life was measured in one study (Tulloch et al., 2021), and self-efficacy was measured in one study (Gortner et al., 1988). In 4 out of 11 studies the primary outcome pertained to marital functioning (Gortner et al., 1988; Stewart et al., 2001; Daugherty et al., 2002; Tulloch et al., 2021). In particular, social support was the primary outcome in the qualitative interview studies of Stewart et al. (2001) and Daugherty et al. (2002). In the study by Gortner et al. (1988) family functioning was quantitatively assessed. Last, in one study (Tulloch et al., 2021) the primary outcomes were relationship quality and couple satisfaction.

Regarding the partner’s primary outcomes: anxiety was measured in 4 studies (Thompson, 1989; Johnston et al., 1999; Hartford et al., 2002; Tulloch et al., 2021), satisfaction with care was measured in 3 studies (Johnston et al., 1999; Daugherty et al., 2002; Dinesen et al., 2019), depression was measured in 2 studies (Johnston et al., 1999; Tulloch et al., 2021), mood states were measured in one study (Gortner et al., 1988), quality of life was measured in one study (Tulloch et al., 2021), disease knowledge was measured in one study (Johnston et al., 1999). In 4 studies (Gortner et al., 1988; Stewart et al., 2001; Daugherty et al., 2002; Tulloch et al., 2021) the primary outcome pertained to marital functioning. In particular, social support was the primary outcome in the qualitative interview studies of Stewart et al. (2001) and Daugherty et al. (2002). In the study by Gortner et al. (1988) family functioning was quantitatively assessed. Last, relationship quality and couple satisfaction were measured in the study by Tulloch et al. (2021).

Regarding the secondary outcomes, in the study by Sher et al. (2014) patient marital satisfaction was measured. One study assessed (Lenz and Perkins, 2000) the level of the partner’s physical status. One study assessed (Lenz and Perkins, 2000) the level of partner depression. Both patient and partner satisfaction with care was also assessed in one study (Tulloch et al., 2021).

#### 3.4.3. Effects of the intervention across time-points

Results from 5 out of the 11 included studies showed that couple-based interventions were more effective than TAU and/or educational programs in increasing patient outcomes. In particular, self-efficacy at 3-month follow-up (Gortner et al., 1988), disease knowledge at 12-month follow-up (Johnston et al., 1999), physical activity at 18-month follow-up (Sher et al., 2014), satisfaction with care (Johnston et al., 1999; Lenz and Perkins, 2000) at 2- and 3-month follow-ups, respectively. Results from 4 out of the 11 included studies showed that couple-based interventions were more effective than TAU and/or educational programs in decreasing patient anxiety at 5-days from discharge (Thompson, 1989), and at 1- (Thompson, 1989), 3- (Thompson, 1989), 6- (Thompson, 1989), 12-month (Johnston et al., 1999) follow-ups. Furthermore, significant decreases in patient depression at 3-month follow-up (Thompson, 1989), blood pressure at 6-month follow-up (Dracup et al., 1984), and patient-perceived disability at 12-month follow-ups (Johnston et al., 1999) were observed. Regarding the 4 interventions that did not include a CG, only the study by Tulloch et al. (2021) had a quantitative approach and showed a significant increase in the quality of life and dyadic adjustment of the patients, and a significant decrease in their partners’ depression, dyadic adjustment, and couple satisfaction at a 2-month follow-up.

Results from 1 out of the 11 included studies showed that couple-based interventions were more effective than TAU and/or educational programs in increasing partner satisfaction with care at 2-month follow-up (Johnston et al., 1999) and disease knowledge at 12-month follow-up (Johnston et al., 1999). A significant decrease in the partner’s anxiety is observed at 5 days from discharge (Thompson, 1989), 3- (Thompson, 1989), 6- (Thompson, 1989), and 12 months (Hartford et al., 2002) follow-ups.

No significant differences were found for depressive or anxiety symptoms at the 6-month follow-up (Dracup et al., 1984), and for physical exercise level (Sher et al., 2014); there was a significant effect of couple treatment on the increased physical activity and acceleration of treatment over time, but there were no condition effects for adherence to medications and nutritional outcomes.

Notably, significant between-group differences favoring couple-based interventions were mostly observed in the short term, and results were not always maintained over time.

## 4. Discussion

This paper explores the existing literature on couple-based interventions for cardiac patients.

Although evidence increasingly supports the dyadic influence that coping with cardiac illness has on cardiac patients and their partners (Cook and Kenny, 2005), findings reveal that couple-based interventions have received little attention in the literature. Moreover, psychological interventions widely vary in terms of the type of intervention, format (group or individual, phone or in person), number of sessions and duration, and personnel involved across the selected records. In addition, the psychological strategies of the interventions are mostly not comprehensively detailed. This makes it difficult to explore which of them had a specific impact on the dyad’s outcomes and prevent effective studies’ replication. Most of the contributions also lack adequate details on the training of the providers, the contents of the interventions, and the theoretical models on which they were based.

Moreover, we could not assume that couple-based interventions are more or less effective than individual ones, since only 2 out of the 11 studies included in this review (Dracup et al., 1984; Sher et al., 2014) compared a couple-based approach with a patient-only approach.

Regarding the type of intervention, two different types of couple-based interventions can be distinguished. The first class of interventions can be labeled as “partner-assisted”–since the partner acts as the patient’s therapist or coach. These interventions often follow a cognitive-behavioral framework, and require specific tasks to be completed outside the treatment sessions. The treatment plan is supported by the couple’s relationship - but does not focus on it–and does not imply the presence of relational difficulties. This type of couple-based intervention is used in 6 out of the 11 included studies (Dracup et al., 1984; Thompson, 1989; Johnston et al., 1999; Daugherty et al., 2002; Hartford et al., 2002; Dinesen et al., 2019).

A second group of couple interventions–used in 5 out of 11 studies (Gortner et al., 1988; Lenz and Perkins, 2000; Stewart et al., 2001; Sher et al., 2014; Tulloch et al., 2021)–focuses on how a couple interacts in scenarios associated with the individual’s disease. These “disorder-specific” interventions consider the couple’s relationships as a variable potentially affecting either the disorder or the treatment.

No study included in this review implemented couple therapy used with the intent of assisting the individual during the treatment based on the assumption that the functioning of the couple contributes in a broad sense to the development or maintenance of their symptoms.

Overall, couple-based interventions have been shown to have only a modest impact on patients’ outcomes including quality of life, psychological distress, level of physical activity, blood pressure, self-efficacy, disease knowledge, satisfaction with care, and dyadic adjustment.

Partners showed improved perceived psychological distress, disease knowledge, and satisfaction with care; and increased dyadic adjustment scores and couple satisfaction at the relational level.

However, although partners are involved in the treatment, the strategies and the outcomes of the studies are mostly focused on the patient. In fact, as previously mentioned also in other studies (e.g., Rapelli et al., 2022, 2023), it is recommended that relational variables be targeted for interventions.

These findings coupled with those of previous systematic reviews documenting the benefit of couple interventions for patients with chronic diseases (Hartmann et al., 2010; Martire et al., 2010) including cardiac illness (Reid et al., 2013).

However, our results need to be interpreted with caution due to the limitations of the included studies.

This review has some limitations, among them not having considered the assessment of methodological quality, but this limitation is pertinent to the objectives of the scoping review and the heterogeneity of the included studies. Furthermore, it is also worth noting that it was decided to exclude gray literature from the study. This may have affected the validity of the study, but gray literature is not usually subjected to a rigorous review process.

As a strength, we can recognize the use of the TIDieR and Consort10 checklists as useful tools for the replicability of studies since they provide a summary of the proposed interventions with an examination of the limitations and criticalities of the studies themselves. In fact, the present scoping review offers significant information that may guide the design of future research and interventions aimed at improving the efficacy and effectiveness of couple-based interventions for cardiac patients. Specifically, we reported guidelines for research and interventions in the next section.

## 5. Future research and clinical directions

For future research, given the limitations of the included studies and in order to determine which psychological interventions are most effective, a large, adequately powered, trial assessing psychological interventions for patients alone, compared with psychological interventions for patients and partners, could be recommended. A variety of variables might be investigated, including the timing of the intervention’s start, its intensity, and duration, while also taking into account a clear definition of intervention, its form of delivery, its content, and the type, education, and experience of the therapist. Furthermore, other information lacking in the included studies was the attrition rate and factors related to participation/non-participation. In fact, according to a recent study (Savioni et al., 2022) future psychological interventions may employ *ad hoc* tools to take into consideration participants’ reasons for non-participation/dropout that often are linked to factors related to intervention commitment and its interference with daily life.

From a clinical point of view, according to the results of this scoping review, more interventions targeted at relational variables are needed. This also means involving the partner in the treatment to increase knowledge of reciprocal needs and activate dyadic resources. The partner would become more aware of the treatment process, more conscious about individual and relational aspects that can influence the patient and the couple’s relationship, and consequently, more involved in the engagement process (Graffigna et al., 2017). Moreover, behavioral modification programs may benefit both the targeted and the non-targeted member of the couple by reducing cardiovascular risk factors in both partners through a virtuous process so one motivates the other since often partners share the same unhealthy diet and inadequate physical activity of patients (Shiffman et al., 2020).

In addition, in the context of chronic illness, it is demonstrated that a group format facilitates the expression and sharing of emotions of both members of the dyad (Saita et al., 2014, 2016) and is cheaper in terms of time and resources compared with individual sessions (Saita et al., 2014).

The use of digital tools might also ensure greater adherence to behavioral changes and support the emotional state of both patients and their partners outside the clinical settings (Graffigna et al., 2017; Dinesen et al., 2019; Bertuzzi et al., 2022). Specifically, telemedicine could be useful to assess caregivers’ burden and to offer specific psychological support to those partners who experience adverse outcomes (Semonella et al., 2022, 2023).

## 6. Conclusion

The available literature on couple-based intervention for cardiac patients is scarce and inconsistent, and mostly focused on the outcomes of the sufferers. More research also considering the dyadic component of the intervention and the specific effect of a given program on informal caregivers is urgently needed. Indeed, in the context of chronic diseases, a fundamental role in supporting the patients is played by their informal caregivers, i.e., those who provide unpaid care to their loved ones (Rapelli et al., 2022, 2023; Semonella et al., 2023).

Caring for a significant other can be a rewarding experience, but due to a lack of time and energy, or financial, emotional, and social strains, it can also turn out to be an overwhelming responsibility for caregivers (Donato et al., 2020; Rapelli et al., 2020a; Bertuzzi et al., 2021).

Therefore, it is important to ensure the wellbeing of partners of individuals with cardiovascular disease, and adequately support them with tailored and integrated healthcare actions within the context of cardiac rehabilitation and through the use of telemedicine.

## Author contributions

GR, EG, CT, GP, and GC contributed to the development of the study, analysis of the results, and writing of the manuscript. AM, GV, and CF revised the manuscript, reviewed methodological as well as clinical issues, and further edited the manuscript. All authors contributed to the article and approved the submitted version.

## References

[B1] Bandura A. (1986). The explanatory and predictive scope of self-efficacy theory. *J. Soc. Clin. Psychol.* 4 359–373. 10.1521/jscp.1986.4.3.359

[B2] Baucom D. H. Shoham V. Mueser K. T. Daiuto A. D. Stickle T. R. (1998). Empirically supported couple and family interventions for marital distress and adult mental health problems. *J. Consult. Clin. Psychol.* 66 53–88. 10.1037/0022-006X.66.1.53 9489262

[B3] Beasley C. C. Ager R. (2019). Emotionally focused couples therapy: A systematic review of its effectiveness over the past 19 years. *J. Evid. Based Soc. Work* 16 144–159. 10.1080/23761407.2018.1563013 30605013

[B4] Bertoni A. Donato S. Graffigna G. Barello S. Parise M. (2015). Engaged patients, engaged partnerships: Singles and partners dealing with an acute cardiac event. *Psychol. Health Med.* 20 505–517. 10.1080/13548506.2014.969746 25506636

[B5] Bertoni A. Rapelli G. Parise M. Pagani A. F. Donato S. (2022). “Cardiotoxic” and “cardioprotective” partner support for patient activation and distress: Are two better than one? *Fam. Relat.* 72, 1335–1350. 10.1111/fare.12694

[B6] Bertuzzi V. Semonella M. Bruno D. Manna C. Edbrook-Childs J. Giusti E. M. (2021). Psychological support interventions for healthcare providers and informal caregivers during the COVID-19 pandemic: A systematic review of the literature. *Int. J. Environ. Res. Public Health* 18:6939. 10.3390/ijerph18136939 PMC8297206 34203529

[B7] Bertuzzi V. Semonella M. Castelnuovo G. Andersson G. Pietrabissa G. (2022). Synthesizing stakeholders perspectives on online psychological interventions to improve the mental health of the italian population during the COVID-19 pandemic: An online survey study. *Int. J. Environ. Res. Public Health* 19 7008. 10.3390/ijerph19127008 PMC9222987 35742257

[B8] Bloom J. R. (1990). The relationship of social support and health. *Soc. Sci. Med.* 30 635–637. 2309140 10.1016/0277-9536(90)90162-l

[B9] Bouchard K. Greenman P. S. Pipe A. Johnson S. M. Tulloch H. (2019). Reducing caregiver distress and cardiovascular risk: A focus on caregiver-patient relationship quality. *Can. J. Cardiol.* 35 1409–1411. 10.1016/j.cjca.2019.05.007 31515084

[B10] Bowlby J. (1969). Disruption of affectional bonds and its effects on behavior. *Canadas Ment. Health Suppl.* 59:12.

[B11] Bowlby J. (1973). *Attachment and loss. Volume II. Separation, anxiety and anger.* New York, NY: Basic Books, 429.

[B12] Breuer N. Sender A. Daneck L. Mentschke L. Leuteritz K. Friedrich M. (2017). How do young adults with cancer perceive social support? A qualitative study. *J. Psychos. Oncol.* 35 292–308. 10.1080/07347332.2017.1289290 28145814

[B13] Cobb S. (1976). Social support as a moderator of life stress. *Psychosom. Med.* 38 300–314. 981490 10.1097/00006842-197609000-00003

[B14] Cook W. L. Kenny D. A. (2005). The actor–partner interdependence model: A model of bidirectional effects in developmental studies. *Int. J. Behav. Dev.* 29 101–109. 10.1080/01650250444000

[B15] Daugherty J. Saarmann L. Riegel B. Sornborger K. Moser D. (2002). Can we talk? Developing a social support nursing intervention for couples. *Clin. Nurse Special.* 16 211–218. 10.1097/00002800-200207000-00011 12172491

[B16] Deci E. L. Ryan R. M. (2000). The” what” and” why” of goal pursuits: Human needs and the self-determination of behavior. *Psychol. Inq.* 11 227–268. 10.1207/S15327965PLI1104_01

[B17] Dinesen B. Nielsen G. Andreasen J. J. Spindler H. (2019). Integration of rehabilitation activities into everyday life through telerehabilitation: Qualitative study of cardiac patients and their partners. *J. Med. Internet Res.* 21:e13281. 10.2196/13281 30985284 PMC6487348

[B18] DiPietro L. Caspersen C. J. Ostfeld A. M. Nadel E. R. (1993). A survey for assessing physical activity among older adults. *Med. Sci. Sports Exerc.* 25 628–642. 8492692

[B19] Donato S. Iafrate R. Bertoni A. M. M. Rapelli G. (2020). “Partner support,” in *Encyclopedia of quality of life and well-being research*, ed. Maggino F. (Cham: Springer), 1–6. 10.1007/978-3-319-69909-7_2087-2

[B20] Dracup K. A. Meleis A. I. (1982). Compliance: An interactionist approach. *Nurs. Res.* 31 31–36. 6922458

[B21] Dracup K. Meleis A. Clarck S. Clyburn A. Shields L. Staley M. (1984). Group counseling in cardiac rehabilitation: Effect on patient compliance. *Patient Educ. Counsel.* 6 169–177. 10.1016/0738-3991(84)90053-3 10269521

[B22] Endler N. S. Parker J. D. (1990). Stress and anxiety: Conceptual and assessment issues. *Stress Med.* 6 243–248. 10.1002/smi.2460060310

[B23] Fait K. Vilchinsky N. Dekel R. Levi N. Hod H. Matetzky S. (2018). Cardiac-diseaseinduced PTSD and Fear of illness progression: Capturing the unique nature of disease related PTSD. *Gen. Hosp. Psychiatry* 53 131–138. 10.1016/j.genhosppsych.2018.02.011 29779574

[B24] Fiske V. Coyne J. C. Smith D. A. (1991). Couples coping with myocardial infarction: An empirical reconsideration of the role of overprotectiveness. *J. Fam. Psychol.* 5 4–20. 10.1037/0893-3200.5.1.4

[B25] Folkman S. (1984). Personal control and stress and coping processes: A theoretical analysis. *J. Pers. Soc. Psychol.* 46 839–852. 10.1037/0022-3514.46.4.839 6737195

[B26] Funk J. L. Rogge R. (2007). *The couples satisfaction index (CSI).* Michigan: Fetzer Institute.

[B27] George-Levi S. Vilchinsky N. Tolmacz R. Khaskiaa A. Mosseri M. Hod H. (2016). “It takes two to take”: Caregiving style, relational entitlement, and medication adherence. *J. Fam. Psychol.* 30 743–751. 10.1037/fam0000203 27513287

[B28] Gill R. Murkin J. M. (1996). Neuropsychologic dysfunction after cardiac surgery: What is the problem? *J. Cardiothorac. Vasc. Anesth.* 10 91–98. 10.1016/S1053-0770(96)80183-2 8634392

[B29] Golan M. Vilchinsky N. (2023). It takes two hearts to cope with an artificial one: The necessity of applying a dyadic approach in the context of left ventricular assist device transplantation—Opinion paper. *Front. Psychol.* 14:1215917. 10.3389/fpsyg.2023.1215917 37575443 PMC10412923

[B30] Golan M. Vilchinsky N. Wolf H. Abuhazira M. Ben-Gal T. Naimark A. (2023). Couples’ coping strategies with left ventricular assist device implantation: A qualitative dyadic study. *Qual. Health Res.* 33 741–752. 10.1177/10497323231168580 37218172

[B31] Gortner S. R. Gilliss C. L. Shinn J. A. Sparacino P. A. Rankin S. Leavitt M. (1988). Improving recovery following cardiac surgery: A randomized clinical trial. *J. Adv. Nurs.* 13 649–661. 10.1111/j.1365-2648.1988.tb01459.x 3066803

[B32] Graffigna G. Barello S. Riva G. Savarese M. Menichetti J. Castelnuovo G. (2017). Fertilizing a patient engagement ecosystem to innovate healthcare: Toward the first Italian Consensus conference on patient engagement. *Front. Psychol.* 8:812. 10.3389/fpsyg.2017.00812 28634455 PMC5460315

[B33] Hartford K. Wong C. Zakaria D. (2002). Randomized controlled trial of a telephone intervention by nurses to provide information and support to patients and their partners after elective coronary artery bypass graft surgery: Effects of anxiety. *Heart Lung* 31 199–206. 10.1067/mhl.2002.122942 12011810

[B34] Hartmann M. Bäzner E. Wild B. Eisler I. Herzog W. (2010). Effects of interventions involving the family in the treatment of adult patients with chronic physical diseases: A meta-analysis. *Psychother. Psychosom.* 79 136–148. 10.1159/000286958 20185970

[B35] Hoffmann T. C. Glasziou P. P. Boutron I. Milne R. Perera R. Moher D. (2014). Better reporting of interventions: Template for intervention description and replication (TIDieR) checklist and guide. *BMJ* 348: g1687. 10.1136/bmj.g1687 24609605

[B36] Huang X. Lin J. Demner-Fushman D. (2006). “Evaluation of PICO as a knowledge representation for clinical questions,” in *Proceedings of the AMIA annual symposium*, Vol. 2006 (Bethesda, MD: American Medical Informatics Association), 359. PMC1839740 17238363

[B37] Jacox A. K. Bausell R. B. Mahrenholz D. M. (1997). Patient satisfaction with nursing care in hospitals. *Outcomes Manag. Nurs. Pract.* 1 20–28. 9432439

[B38] Johnston M. Foulkes J. Johnston D. W. Pollard B. Gudmundsdottir H. (1999). Impact on patients and partners of inpatient and extended cardiac counseling and rehabilitation: A controlled trial. *Psychosom. Med.* 61 225–233. 10204976 10.1097/00006842-199903000-00015

[B39] Khan K. S. Kunz R. Kleijnen J. Antes G. (2003). Five steps to conducting a systematic review. *J. R. Soc. Med.* 96 118–121. 10.1258/jrsm.96.3.118 12612111 PMC539417

[B40] Lave J. Wenger E. (1991). “Learning in doing: Social, cognitive, and computational perspectives,” in *Situated learning: Legitimate peripheral participation* Vol. 10 Cambridge: Cambridge University Press, 109–155.

[B41] Lazarus R. S. (1974). Psychological stress and coping in adaptation and illness. *Int. J. Psychiatry Med.* 5 321–333. 10.2190/T43T-84P3-QDUR-7RTP 4618837

[B42] Leifheit-Limson E. C. Kasl S. V. Lin H. Buchanan D. M. Peterson P. N. Spertus J. A. (2012). Adherence to risk factor management instructions after acute myocardial infarction: The role of emotional support and depressive symptoms. *Ann. Behav. Med.* 43 198–207. 10.1007/s12160-011-9311-z 22037964 PMC3374717

[B43] Lenz E. R. Perkins S. (2000). Coronary artery bypass graft surgery patients and their family member caregivers: Outcomes of a family-focused staged psychoeducational intervention. *Appl. Nurs. Res.* 13 142–150. 10.1053/apnr.2000.7655 10960998

[B44] Locke H. J. Wallace K. M. (1959). Short marital-adjustment and prediction tests: Their reliability and validity. *Marriage Fam. Living* 21 251–255.

[B45] Luttik M. L. Blaauwbroek A. Dijker A. Jaarsma T. (2007). Living with heart failure: Partner perspectives. *J. Cardiovasc. Nurs.* 22 131–137. 10.1016/j.aucc.2007.05.004 17318039

[B46] Maeda U. Shen B. J. Schwarz E. R. Farrell K. A. Mallon S. (2013). Self-efficacy mediates the associations of social support and depression with treatment adherence in heart failure patients. *Int. J. Behav. Med.* 20 88–96. 10.1007/s12529-011-9215-0 22212607

[B47] Martire L. M. Schulz R. Helgeson V. S. Small B. J. Saghafi E. M. (2010). Review and meta-analysis of couple-oriented interventions for chronic illness. *Ann. Behav. Med.* 40 325–342. 10.1007/s12160-010-9216-2 20697859 PMC4101802

[B48] McCubbin H. I. Patterson J. M. (2014). “The family stress process: The double ABCX model of adjustment and adaptation,” in *Social stress and the family*, McCubbin H. I. M. Sussman M. S. B. Patterson J. M. (Milton Park: Routledge), 7–37.

[B49] McCubbin H. I. Comeau J. K. Harkins J. A. (1987). “Family inventory of resources for management,” in *Family assessment inventories for research and practice*, (New York, NY: Oxford University), 145–160.

[B50] McNair D. M. Lorr M. Droppleman L. F. (1971). *Profile of mood states.* San Diego, CA: Educationai and Industrial Testing SeMce.

[B51] Nelson E. C. Wasson J. H. Kirk J. W. (1987). Assessment of function in routine clinical practice: Description of the COOP chart method and preliminary findings. *J. Chron. Dis.* 49(Suppl. 1) 55S–63S. 10.1016/s0021-9681(87)80033-4 3597698

[B52] Patrick D. L. Peach H. (1989). “A socio-medical approach to disablement,” in *Disablement in the Community*, eds Patrick D. L. Peach H. (Oxford: Oxford University Press), 1–18.

[B53] Prochaska J. O. DiClemente C. C. (2005). The transtheoretical approach. *Handb. Psychother. Integr.* 2 147–171.

[B54] Radloff L. S. (1977). The CES-D scale: A self-report depression scale for research in the general population. *Appl. Psychol. Meas.* 1 385–401. 10.1177/014662167700100306 26918431

[B55] Randall G. Molloy G. J. Steptoe A. (2009). The impact of an acute cardiac event on the partners of patients: A systematic review. *Health Psychol. Rev.* 3 1–84. 10.1080/17437190902984919

[B56] Rankin-Esquer L. A. Deeter A. K. Froeliche E. Taylor C. B. (2000). Coronary heart disease: Intervention for intimate relationship issues. *Cogn. Behav. Pract.* 7 212–220. 10.1016/S1077-7229(00)80034-6

[B57] Rapelli G. Donato S. Bertoni A. (2020a). Il partner del paziente cardiologico: Chi sostiene chi? *Psicol. Della Salute* 1 92–103. 10.3280/PDS2020-001008

[B58] Rapelli G. Donato S. Bertoni A. Spatola C. Pagani A. F. Parise M. (2020b). The combined effect of psychological and relational aspects on cardiac patient activation. *J. Clin. Psychol. Med. Settings* 27 783–794. 10.1007/s10880-019-09670-y 31630348

[B59] Rapelli G. Donato S. Pagani A. F. Parise M. Iafrate R. Pietrabissa G. (2021). The association between cardiac illness-related distress and partner support: The moderating role of dyadic coping. *Front. Psychol.* 12:106. 10.3389/fpsyg.2021.624095 33679540 PMC7925924

[B60] Rapelli G. Donato S. Parise M. Pagani A. F. Castelnuovo G. Pietrabissa G. (2022). Yes, i can (With You)! Dyadic coping and self-management outcomes in cardiovascular disease: The mediating role of health self-efficacy. *Health Soc. Care Commun.* 30:e2604–e2617. 10.1111/hsc.13704 34985787

[B61] Rapelli G. Giusti E. M. Donato S. Parise M. Pagani A. F. Pietrabissa G. (2023). The heart in a bag”: The lived experience of patient-caregiver dyads with Left Ventricular Assist Device (LVAD) during cardiac rehabilitation. *Front. Psychol.* 14:913. 10.3389/fpsyg.2023.1116739 37089738 PMC10114412

[B62] Reid J. Ski C. F. Thompson D. R. (2013). Psychological interventions for patients with coronary heart disease and their partners: A systematic review. *PLoS one* 8:e73459. 10.1371/journal.pone.0073459 24039950 PMC3764157

[B63] Roger V. L. Sidney S. Fairchild A. L. Howard V. J. Labarthe D. R. Shay C. M. (2020). Recommendations for cardiovascular health and disease surveillance for 2030 and beyond: A policy statement from the American Heart Association. *Circulation* 141 e104–e119. 10.1161/CIR.0000000000000756 31992050

[B64] Saita E. Acquati C. Molgora S. (2016). Promoting patient and caregiver engagement to care in cancer. *Front. Psychol.* 7:1660. 10.3389/fpsyg.2016.01660 27826279 PMC5079095

[B65] Saita E. Molgora S. Acquati C. (2014). Development and evaluation of the cancer dyads group intervention: Preliminary findings. *J. Psychos. Oncol.* 32 647–664. 10.1080/07347332.2014.955242 25229893

[B66] Savioni L. Triberti S. Durosini I. Sebri V. Pravettoni G. (2022). Cancer patients’ participation and commitment to psychological interventions: A scoping review. *Psychol. Health* 37 1022–1055. 33966548 10.1080/08870446.2021.1916494

[B67] Schulz K. F. Altman D. G. Moher D. Fergusson D. (2010). CONSORT 2010 changes and testing blindness in RCTs. *Lancet* 375 1144–1146. 10.1016/S0140-6736(10)60413-8 20338625

[B68] Sebri V. Mazzoni D. Triberti S. Pravettoni G. (2021). The impact of unsupportive social support on the injured self in breast cancer patients. *Front. Psychol.* 12:722211. 10.3389/fpsyg.2021.722211 34616337 PMC8488137

[B69] Semonella M. Andersson G. Dekel R. Pietrabissa G. Vilchinsky N. (2022). Making a virtue out of necessity: COVID-19 as a catalyst for applying Internet-based psychological interventions for informal caregivers. *Front. Psychol.* 13:856016. 10.3389/fpsyg.2022.856016 35465576 PMC9022647

[B70] Semonella M. Bertuzzi V. Dekel R. Andersson G. Pietrabissa G. Vilchinsky N. (2023). Applying dyadic digital psychological interventions for reducing caregiver burden in the illness context: A systematic review and a meta-analysis protocol. *BMJ Open* 13:e070279. 10.1136/bmjopen-2022-070279 PMC10173984 37164463

[B71] Sher T. Braun L. Domas A. Bellg A. Baucom D. H. Houle T. T. (2014). The partners for life program: A couples approach to cardiac risk reduction. *Fam. Process* 53 131–149. 10.1111/famp.12061 24495204 PMC3959575

[B72] Shiffman D. Louie J. Z. Devlin J. J. Rowland C. M. Mora S. (2020). Concordance of cardiovascular risk factors and behaviors in a multiethnic US nationwide cohort of married couples and domestic partners. *JAMA Netw. Open* 3:e2022119. 10.1001/jamanetworkopen.2020.22119 PMC7588939 33104207

[B73] Smilkstein G. (1978). The family APGAR: A proposal for a family function test and its use by physicians. *J. Fam. Pract.* 6 1231–1239. 660126

[B74] Smith V. Devane D. Begley C. M. Clarke M. (2011). Methodology in conducting a systematic review of systematic reviews of healthcare interventions. *BMC Med. Res. Methodol.* 11:15. 10.1186/1471-2288-11-15 21291558 PMC3039637

[B75] Sokoreli I. De Vries J. J. G. Pauws S. C. Steyerberg E. W. (2016). Depression and anxiety as predictors of mortality among heart failure patients: Systematic review and meta-analysis. *Heart Fail. Rev.* 21 49–63. 10.1007/s10741-015-9517-4 26572543

[B76] Spanier G. B. (1976). Measuring dyadic adjustment: New scales for assessing the quality of marriage and similar dyads. *J. Marriage Fam.* 38 15–28.

[B77] Stephens M. A. P. Martire L. M. Cremeans-Smith J. K. Druley J. A. Wojno W. C. (2006). Older women with osteoarthritis and their caregiving husbands: Effects of pain and pain expression on husbands’ well-being and support. *Rehabil. Psychol.* 51 3–12. 10.1037/0090-5550.51.1.3

[B78] Stewart M. Davidson K. Meade D. Hirth A. Weld-Viscount P. (2001). Group support for couples coping with a cardiac condition. *J. Adv. Nurs.* 33 190–199. 11168702 10.1046/j.1365-2648.2001.01652.x

[B79] Thompson D. R. (1989). A randomized controlled trial of in-hospital nursing support for first time myocardial infarction patients and their partners: Effects on anxiety and depression. *J. Adv. Nurs.* 14 291–297. 10.1111/j.1365-2648.1989.tb03416.x 2738227

[B80] Tulloch H. Johnson S. Demidenko N. Clyde M. Bouchard K. Greenman P. S. (2021). An attachment-based intervention for patients with cardiovascular disease and their partners: A proof-of-concept study. *Health Psychol.* 40 909–919. 10.1037/hea0001034 33346674

[B81] Vilchinsky N. Ginzburg K. Fait K. Foa E. B. (2017). Cardiac-disease-induced PTSD (CDI-PTSD): A systematic review. *Clin. Psychol. Rev.* 55 92–106. 10.1016/j.cpr.2017.04.009 28575815

[B82] Wiebe S. A. Johnson S. M. (2016). A review of the research in emotionally focused therapy for couples. *Fam. Process* 55 390–407. 10.1111/famp.12229 27273169

[B83] Zigmond A. S. Snaith R. P. (1983). The hospital anxiety and depression scale. *Acta Psychiatr. Scand.* 67 361–370. 10.1111/j.1600-0447.1983.tb09716.x 6880820

