# Supplementary material for: Psychological couple-oriented interventions for patients with heart disease and their partners: a scoping review and guidelines for future interventions
Source: Front Psychol. 2023 Sep 29;14:1194767. doi: 10.3389/fpsyg.2023.1194767 (PMC10570454; doi:10.3389/fpsyg.2023.1194767)
Supplement: Supplementary file 2 [file Table_2.DOCX]

*Primary outcomes - patient*

***Anxiety.*** The patient’s anxiety was measured in 4 out of 11 studies (Hartford et al., 2002; Johnston et al., 1999; Thompson, 1989; Tulloch et al., 2021) using the Back Anxiety Inventory (BAI) (Hartford et al., 2002), and the Hospital Anxiety and Depression Scale (HADS) (Johnston et al., 1999; Thompson, 1989; Tulloch et al., 2021). Furthermore, Thompson (1989) assessed the level of anxiety employing a visual analog scale (VAS)

In the study by Hartford and colleagues (2002), anxiety assessed at four different times underwent moderate-severe evolution from the baseline until the last assessment where no differences emerged between the patients allocated to the IG and those in the CG.

Johnston and colleagues (1999) reported that IG at one-year follow-up significantly reduced levels of patients’ anxiety compared to CG (TAU). In particular, patients of IG1 (IG1: in-patient) had lower scores than the CG at discharge, and at 2 months, and patients of IG2 (IG2: in-patient + out-patient) had lower levels than the CG at 2-, 6- and 12-months follow-ups. The patients of IG2 had lower anxiety than IG1 at 2- and 6-months follow-ups.

Similarly, Thompson (1989) reported that in all follow-up points and also at the final follow-up at 6-months patients of IG showed significantly lower levels of general anxiety and specific anxiety scales than those reported by patients of CG.

In the pre-post study by Tulloch et al. (2021), patients did not report significant changes in anxiety levels over time.

***Depression.*** The patient’s depression was measured in 4 out of 11 studies (Lenz and Perkins, 2000; Johnston et al., 1999; Thompson, 1989; Tulloch et al., 2021) using the Center for Epidemiologic Studies-Depression (CES-D) in the study by Lenz and Perkins (2000), The Hospital Anxiety and Depression Scale (HADS) in two studies (Johnston et al., 1999; Thompson, 1989).

Lenz and Perkins (2000) reported that patients of IG had higher levels of depressive symptoms at 3-month follow-up but these differences were non-significant.

Johnston and colleagues (1999) reported that IG at one-year follow-up reduced levels of patients’ depression compared to CG (TAU). In particular, patients of IG1 (IG1: in-patient) who received in-patient counseling intervention had lower levels of depression than the CG at 6 months; whereas the patients of IG2 who received in-patient + out-patient counseling intervention had lower levels than the CG at 2- and 6-months follow-up.

Thompson (1989) reported that patients in IG at 3 months follow-up reported significantly lower levels of depression than patients in CG, but at the final follow-up at 6-month were no reported statistically significant differences.

In the pre-post study by Tulloch et al. (2021), patients reported a significant decrease in depression levels over time.

***Emotional state.*** The Profile of Mood States (POMS) was used in one study (Gortner et al., 1988) and revealed no statistically significant differences in the emotional state of patients in the IG compared with those in the CG at 6-month follow-up.

***Quality of life.*** The patient’s quality of life was measured in the pre-post study by Tulloch et al. (2021) using the mental component (QoL-MCS) and physical component (QoL-PCS) dimension of the Medical Outcomes Survey Short Form-36 (SF-36 V.1). Patients reported significant increases in the levels of QoL-MCS over time. But no significant changes were noted for QoL-PCS.

***Knowledge of disease and treatment.*** The patient’s knowledge of his/her behavioral changes in terms of cardiac risk-factors reduction (knowledge of the disease and rehabilitation process about pharmacological adherence, diet, smoking, blood pressure, body weight, diet, adhesion to drugs and adherence to treatment in long-term) was measured in 3 out of 11 studies (Dracup et al., 1984; Johnston et al., 1999; Sher et al., 2014) using ad hoc items (Johnston et al., 1999), measuring weight loss and physical measurements (e.g., BMI; hours exercised per week; systolic and diastolic blood pressures) in two studies (Dracup et al., 1984; Sher et al., 2014), and with a computerized system for Adhesion to drugs (computerized Medication Event Monitoring System; MEMS) in the study by Sher et al. (2014).

The results of the study by Dracup and colleagues (1984) support the positive effects of counseling intervention aimed at increasing compliance by reducing specific cardiovascular risk-factors such as smoking, blood pressure, body weight, and sedentary lifestyle. However, the comparison of IG and CG indicated that participation in a cardiac rehabilitation program is not, in itself, sufficient to influence long-term compliance. In particular, smoking has not been significantly different over time between groups for both patients and partners. The IG2 (only for patient) showed the greatest decrease over time in body fat than IG1 (couple-based intervention). Patients in both IGs (IG1 and IG2) showed lower blood pressure than TAU at 6-month follow-up.

Again, the largest decrease was in IG2. Changes in weekly exercise level were not significantly different among groups, although the highest compliance was reported by patients of IG1. However, patients who participated in the rehabilitation program with their spouses (IG1) showed greater compliance than the CG.

In the study by Johnston et al. (1999) patients of IG1 (in-patient) and IG2 (in-patient + out-patient) reported higher levels of knowledge than CG at one-year follow-up: specifically, patients report higher levels of correct information and ideas and lower levels of uncertain information. On satisfaction with care IG1 and IG2 have higher levels than CG.

In the study by Sher et al. (2014) patients of IG (couple-based intervention) and those of CG (individual intervention) did not show significant changes in medication adherence, but patients in CG showed a 9% relative decrease across time. Furthermore, there were no significant effects on nutritional outcomes and weight loss variables (such as BMI) across time or between treatment conditions.

***Physical status.*** In 3 out of 11 studies (Johnston et al., 1999; Lenz and Perkins, 2000; Sher et al., 2014) the level of the patient’s physical status was measured with Functional Limitations Profile (FLP) which measured limitations and return to normal activities in the study by Johnston et al. (1999) and with COOP Functional health status (overall) in the study by Lenz and Perkins (2000). Also in the study by Sher et al. (2014), physical activity was measured with the YALE physical activity survey (YPAS).

In the study by Johnston et al. (1999) patients of IG1 had significantly lower levels of disability measured with FLP than patients of CG (TAU) at 12-month follow-up one.

Lenz and Perkins (2000) reported small non-significant lower levels of disability in the patients of IG compared with the CG at 3-months follow-up.

In the study by Sher et al. (2014) patient physical activity increased significantly over time in both IG (Couple condition) and CG (Patient individual condition). In particular, patients in CG changed physical activity at a greater overall rate initially, but the rate decelerated over time; in contrast, patients in the IG showed slow but steady improvement in physical activity across treatment and follow-up periods with no flattening out or deterioration, surpassing the control patients at around week 14.

***Satisfaction with care.*** In 4 studies (Daugherty et al., 2002; Dinesen et al., 2019; Johnston et al., 1999; Lenz and Perkins, 2000) the primary outcome was satisfaction with care measured with ad hoc items.

In the qualitative interview study of Daugherty and colleagues (2002), the intervention was perceived as effective for patients.

In the qualitative study intervention of Dinesen et al. (2019). Patients judged telerehabilitation to be a useful intervention for the education of patients in the rehabilitation process because it provided relevant information about heart disease, symptoms, and lifestyle changes. Telerehabilitation has also fostered patient autonomy as they are more involved in making personal decisions and more motivated by their partners to continue lifestyle changes.

In the study by Johnston et al. (1999) patients of IG1 (in-patient) and IG2 (in-patient + out-patient) had higher levels of satisfaction with care than participants of CG at a 2-months follow-up.

Lenz and Perkins (2000) reported higher levels of satisfaction with nursing care in patients of IG than the CG at 3-month follow-up, but the difference was not statistically significant.

***Self-efficacy.*** In one study, the primary outcome was self-efficacy (Gortner et al., 1988) measured with ad hoc items. Gortner and colleagues (1988) find significant differences in the perception of self-efficacy: patients in IG perceived at 3-month a greater self-efficacy than patients of CG. However, at 6-month follow-up the differences were not statistically significant.

***Marital functioning and relational variables.*** In 4 out of 11 studies (Daugherty et al., 2002; Gortner et al., 1988; Stewart et al., 2001; Tulloch et al., 2021) the primary outcome pertained to marital functioning. In particular, social support was the primary outcome in the qualitative interview studies of Daugherty et al. (2002) and Stewart et al. (2001). In the study by Gortner and colleagues (1988) family functioning was measured using The family APGAR (Adaptability, Partnership, Growth, Affection, and Resolve); the Family Inventory of Resources for Management (FIRM); and for marital satisfaction, the Marital adjustment test (MAS) was used. Also in the study by Tulloch et al. (2021), the primary outcomes were the relationship quality and couple satisfaction measured with the Dyadic Adjustment Scale (DAS) and the Couple Satisfaction Index (CSI) respectively.

From the discussion in focus groups with couples (Daugherty et al., 2002), emerged the importance of the partner’s support and, and how to change the partner’s supportive behavior toward the patient. In fact, they discussed about the negative consequences of a support based on overprotection that limit the patient's autonomous abilities and hostile support, criticizing the patient when he/she communicates the burden of the disease or related psychological distress.

According to the study by Stewart and colleagues (2000), the exchange between participants during the focus group allowed patients to focus on problems about cardiac rehabilitation and cardiac self-management. The intervention greatly improved communication between spouses, mutual understanding, and acceptance within the couple; it promoted better dyadic coping strategies to cope with stressful situation and the marital quality of both partners.

Gortner et al. (1988) reported that for the measures of family functioning (APGAR), family resources (FIRM), and marital satisfaction (MAS) no significant differences were observed between patients of IG and those of CG at 3- and 6-month follow-ups.

In the study by Tulloch et al. (2021) patients showed increased levels of relationship quality measured by the DAS and increased levels of couple satisfaction measured with CSI from pre- to post-intervention.

***Primary outcomes – partner***

***Anxiety.*** The partner’s anxiety was measured in 4 out of 11 studies (Hartford et al., 2002; Johnston et al., 1999; Thompson, 1989; Tulloch et al., 2021) using Back Anxiety Inventory (BAI) in the study by Hartford et al. (2002), The Hospital Anxiety and Depression Scale (HADS) in three studies (Johnston et al., 1999; Thompson, 1989; Tulloch et al., 2021). Furthermore, Thompson (1989) used a VAS scale to assess anxiety.

In the study by Hartford and colleagues (2002), anxiety assessed at four different times underwent moderate-severe evolution from the baseline until the last assessment where no differences in anxiety levels emerged between the partners of IG and the CG.

Johnston and colleagues (1999) reported that IG at one-year follow-up reduced levels of partner anxiety compared to CG (TAU). In particular, partners of IG1 (IG1: in-patient) had lower scores than the CG at discharge, and at 2 months and partners of IG2 (IG2: in-patient + out-patient) had lower levels than the CG at 2-, 6- and 12-months follow-ups. The partners of IG2 had lower anxiety than IG1 at 2- and 6-months follow-ups.

Similarly, Thompson (1989) reported that in all follow-up points and also at the final follow-up at 6-months patients of IG showed significantly lower levels of general anxiety and specifical anxiety scales than those reported by partners of CG.

In the pre-post-study by Tulloch et al. (2021), partners reported a significant decrease in anxiety levels over time.

***Depression.*** The partner’s depression was measured in 2 studies (Johnston et al., 1999; Tulloch et al., 2021) using The Hospital Anxiety and Depression Scale (HADS).

Johnston and colleagues (1999) reported that partners of IG at one-year follow-up showed reduced levels of partners’ depression compared to CG (TAU). In particular, partners of IG1 (IG1: in-patient) who received in-patient counseling intervention had lower levels of depression than the CG at 6 months; whereas the partners of IG2 who received in-patient + out-patient counseling intervention had lower levels than the CG at 2- and 6-months follow-up.

In the pre-post study by Tulloch et al. (2021), partners reported a significant decrease in depression levels over time.

***Emotional state.*** In one study (Gortner et al., 1988) the partner’s emotional state in terms of mood states was measured with the Profile of Mood States (POMS). Results showed that there were no significant differences in emotional state among partners of IG compared with partners of CG at 6-month follow-up.

***Quality of life.*** The partner’s quality of life was measured in the pre-post study by Tulloch et al. (2021) using the validated Medical Outcomes Survey Short Form-36 (SF-36 V.1) distinguished mental component (QoL-MCS) and physical component (QoL-PCS). Partners did not report significant increases in mental and physical quality of life (QoL-MCS and QoL-PCS) over time.

***Knowledge of the disease and the treatment.*** In the study by Johnston et al. (1999) partners of IG1 (in-patient) and IG2 (in-patient + out-patient) reported higher levels of knowledge than CG at one-year follow-up: specifically, partners reported higher levels of correct information and ideas and lower levels of uncertain information.

***Satisfaction with care.*** In 3 studies (Daugherty et al., 2002; Dinesen et al., 2019; Johnston et al., 1999) the primary outcome was the partner’s perception of satisfaction with care measured with ad hoc items.

In the qualitative interview study of Daugherty and colleagues (2002), the intervention was perceived as effective for partners.

In the qualitative study intervention of Dinesen et al. (2019). Partners judged telerehabilitation to be a useful intervention for the education of patients in the rehabilitation process because it provided relevant information about heart disease, symptoms, and lifestyle change. Telerehabilitation has also fostered patient autonomy as they are more involved in making personal decisions and more motivated by their partners to continue lifestyle changes. In fact, spouses have proved to be an important resource of support in everyday life for patients and have evaluated the telematic rehabilitation plan as very useful to facilitate understanding and consistency in the rehabilitation process for both themselves and the patient, thus creating a greater feeling of safety.

Partners of IG1 (in-patient) and IG2 (in-patient + out-patient) in the study by (Johnston et al., 1999) had higher levels of satisfaction with care than partners of CG at 2-month follow-up.

***Marital functioning and relational variables.*** In 4 out of 11 studies (Daugherty et al., 2002; Stewart et al., 2001; Gortner et al., 1988; Tulloch et al., 2021) the primary outcome pertained to marital functioning. In particular, social support was the primary outcome in the qualitative interview studies of Daugherty et al. (2002) and Stewart et al. (2001). In the study by Gortner and colleagues (1988) family functioning was measured using The family APGAR (Adaptability, Partnership, Growth, Affection, and Resolve); the Family Inventory of Resources for Management (FIRM); and for marital satisfaction, the Marital adjustment test (MAS) was used. In the study by Tulloch et al. (2021), the primary outcomes were the relationship quality and couple satisfaction measured with the Dyadic Adjustment Scale (DAS) and the Couple Satisfaction Index (CSI) respectively.

From the discussion in focus groups with couples (Daugherty et al., 2002), emerged the importance of the partner’s support and, and how to change the partner’s supportive behavior toward the patient. In fact, they discussed the negative consequences of a support based on overprotection that limit the patient's autonomous abilities and hostile support, criticizing the patient when he/she communicates the burden of the disease or related psychological distress.

According to the study by Stewart and colleagues (2000), the exchange between participants during the focus group allowed partners to focus on problems about cardiac rehabilitation and cardiac self-management. The intervention greatly improved communication between spouses, mutual understanding, and acceptance within the couple; it promoted better dyadic coping strategies to cope with stressful situation and the marital quality of both partners.

Gortner et al. (1988) reported that for the measures of family functioning (APGAR), family resources (FIRM), and marital satisfaction (MAS) no significant differences were observed between partners of IG and those of CG at 3- and 6 months follow-ups.

In the study by Tulloch et al. (2021), partners showed increased levels of relationship quality measured by the DAS and increased levels of couple satisfaction measured with CSI from pre- to post-intervention.

*Secondary outcomes – patient*

***Marital satisfaction.*** In the study by Sher et al. (2014) marital satisfaction was measured using Dyadic Adjustment Scale (DAS). The overall change in marital satisfaction was small and statistically non-significant over the time of the study in both IG (Couple-based intervention) and CG (Patient individual intervention), and there was no effect of treatment condition on marital satisfaction. In particular, the interaction effect showed that patients in the individual condition who reported lower levels of initial marital satisfaction showed deterioration in marital satisfaction, while non-satisfied participants in the couples treatment showed improvement over time.

*Secondary outcomes – partner*

***Physical status.*** One study assessed (Lenz and Perkins, 2000) the level of partner physical status using the COOP Functional health status (overall). Lenz and Perkins (2000) reported small non-significant lower levels of disability in the partners of IG compared with the CG at 3-months follow-up.

***Depression.*** One study assessed (Lenz and Perkins, 2000) the level of partner depression using the using Center for Epidemiologic Studies-Depression (CES-D). Lenz and Perkins (2000) reported no significant difference between IG and CG, but partners depressive symptoms decreased over time.

*Secondary outcomes – patient and partner*

***Satisfaction with care.*** The participants’ satisfaction with care was assessed in one study (Tulloch et al., 2021) using ad hoc items (ranging from 0 to 5). Participants showed high levels of satisfaction with the intervention overall and for each individual session. Ratings of the overall content, facilitator interactions, usefulness of the session exercises, usefulness of the take-home exercises, and content of the video clips were similarly high.
